# Three dimensional reconstruction to visualize atrial fibrillation activation patterns on curved atrial geometry

**DOI:** 10.1371/journal.pone.0249873

**Published:** 2021-04-09

**Authors:** Ricardo Abad, Orvil Collart, Prasanth Ganesan, A. J. Rogers, Mahmood I. Alhusseini, Miguel Rodrigo, Sanjiv M. Narayan, Wouter-Jan Rappel

**Affiliations:** 1 Stanford Cardiovascular Institute, Stanford University School of Medicine, Stanford, California, United States of America; 2 Universitat Politècnica de València, Valencia, Spain; 3 Department of Physics, UC San Diego, La Jolla, California, United States of America; University of Minnesota, UNITED STATES

## Abstract

**Background:**

The rotational activation created by spiral waves may be a mechanism for atrial fibrillation (AF), yet it is unclear how activation patterns obtained from endocardial baskets are influenced by the 3D geometric curvature of the atrium or ‘unfolding’ into 2D maps. We develop algorithms that can visualize spiral waves and their tip locations on curved atrial geometries. We use these algorithms to quantify differences in AF maps and spiral tip locations between 3D basket reconstructions, projection onto 3D anatomical shells and unfolded 2D surfaces.

**Methods:**

We tested our algorithms in N = 20 patients in whom AF was recorded from 64-pole baskets (Abbott, CA). Phase maps were generated by non-proprietary software to identify the tips of spiral waves, indicated by phase singularities. The number and density of spiral tips were compared in patient-specific 3D shells constructed from the basket, as well as 3D maps from clinical electroanatomic mapping systems and 2D maps.

**Results:**

Patients (59.4±12.7 yrs, 60% M) showed 1.7±0.8 phase singularities/patient, in whom ablation terminated AF in 11/20 patients (55%). There was no difference in the location of phase singularities, between 3D curved surfaces and 2D unfolded surfaces, with a median correlation coefficient between phase singularity density maps of 0.985 (0.978–0.990). No significant impact was noted by phase singularities location in more curved regions or relative to the basket location (p>0.1).

**Conclusions:**

AF maps and phase singularities mapped by endocardial baskets are qualitatively and quantitatively similar whether calculated by 3D phase maps on patient-specific curved atrial geometries or in 2D. Phase maps on patient-specific geometries may be easier to interpret relative to critical structures for ablation planning.

## Introduction

Atrial fibrillation (AF) is the most common cardiac arrhythmia in the United States and is characterized by incoherent electrical activation of the atria [1]. Although it is challenging to map AF with high temporal/spatial resolution, recent studies mapping AF globally using either contact basket electrodes within the atria or electrode arrays on the body surface analyzed by the inverse solution, reveal rotational activation patterns [2–8]. These rotational activation patterns correspond to spiral waves with tip locations that can be temporally and spatially stable or unstable [9–12].

Drug therapy to maintain sinus rhythm is often not successful, and ablation to eliminate critical tissue is an increasingly-used alternative. Pulmonary vein isolation (PVI) is the cornerstone of ablation, designed to isolate tissue near the PVs from the rest of the atria [13]. PVI has a success rate of 40–70%, which is higher than other therapies, but has been difficult to improve by additional ablation [14]. More recently, tip locations of spiral waves have been targeted by ablation [2,7,15–21], resulting in mixed results [22,23] that may improve in subsets of patients treated by targeted ablation PVI [24].

An often-used clinical approach to map these rotational activation patterns is to use contact multipolar catheters [6,25] and analyze recorded electrograms to identify the location of spiral wave tips [26,27]. The data from these catheters is often analyzed and then visualized on unfolded 2D surface representations, and any projection errors can lead to incorrect identification of spiral tip locations. Indeed, a recent study suggested that some variability in AF ablation may result from such inaccurate projections [28].

To address this potential inaccuracy, we developed a new 3D reconstruction approach independent of electroanatomic mapping systems. Using this approach, we tested the hypothesis that phase maps of AF computed directly in 3D space are conserved compared to phase maps computed directly in 2D projections. We analyzed data independently of clinical electroanatomic mapping systems, which may introduce errors of volume reconstruction and accuracy [29,30]. This hypothesis followed our reasoning that 2D and 3D mapping should not qualitatively alter AF maps unless relative electrode locations are changed (e.g. splines are crossed). One analogy is that North-South-East-West information of the Earth is maintained for all depictions. As a consequence, the tip location of a spiral wave should be bounded by the same 4 electrodes in the 2D and the 3D maps. Nevertheless, the size of organized domains or other metrics may vary from 3D and 2D, particularly for the equator versus the poles or at significantly curved regions of the atria.

We tested our hypothesis by creating accurate mathematical tools to examine activation maps directly in 3D from known (X,Y,Z) electrode coordinates rather than projecting to atrial anatomy from clinical electroanatomic systems. We tested this approach in patients undergoing AF ablation guided by basket mapping including those in whom prospective ablation at identified spiral tip locations terminated AF.

## Methods

The study was approved by the Stanford Institutional Review Board (#35346), and all patients provided written informed consent.

### Patient inclusions

We studied 20 patients with AF (70% persistent, defined as patients in whom AF lasted longer than 7 days) recruited for ablation at Stanford University Hospital, Palo Alto, CA, in whom AF was refractory to ≥1 anti-arrhythmic medication. Patients were part of the COMPARE-AF registry (NCT02997254), in whom AF was mapped using 64 pole contact baskets and positional data on electrode position was available to develop novel reconstruction methods.

### Electrophysiological study

Patients were studied in the post-absorptive state. Class I and III anti-arrhythmic medications were discontinued for > 5 half-lives (>30 days for amiodarone). Catheters were advanced to the right atrium (RA), coronary sinus and transseptally to left atrium (LA). Contact basket catheters (FIRMap, Abbott) were positioned in RA then LA for AF mapping, based upon 3-dimensional electroanatomic imaging (NavX, St Jude Medical, Sylmar, CA; or Carto, Biosense-Webster, Diamond Bar, CA). This catheter consists of 8 splines, each with 8 electrodes, totaling 64 electrodes, which cover >70% of each atrium [31]. Within a spline, electrodes are separated by 4–6 mm, and spacing between splines is mostly within 20% of that range [31]. Ablation was guided prospectively at regions of interest identified by a commercial system (RhythmView^™^, Abbott, Inc.) by delivering radiofrequency energy via an irrigated catheter (Thermocool, Biosense-Webster; or Sapphire-Blue, St Jude Medical) at 25–35 watts.

### Data export for analysis

Unipolar electrograms were recorded at 0.05 to 500 Hz bandpass, at 1 kHz sampling with electroanatomic location turned off to reduce electromagnetic interference. We analyzed data from the 4 second time window used clinically to guide ablation. Each data array contained voltage time-series comprising 64 basket electrograms, intracardiac channels such as the coronary sinus, and the 12-lead ECG. Data were exported for analysis from the Bard (LabSystem Pro) or Prucka (GE Cardiolab) electrophysiological recorder. Examples of electrograms are shown in S1 Fig.

### AF mapping method

Since differences in reported AF mechanisms may reflect mapping methods [32], we used only freely available algorithms to construct AF activation patterns [6,33] rather than proprietary methods. The central analysis of AF in this study used a phase-based method. This method has been validated in both animal models [33,34] and in human persistent AF. In the latter, it identified rotational sites of termination by ablation that correlated with clinical mapping methods and identifies sites where localized ablation terminated persistent AF [35,36]. Code and data for this method have been placed online (https://github.com/Rappel-lab/cardiac-codes/tree/main/2d-vs-3d).

Our phase mapping approach has been detailed before [37]. Briefly, it first processes the raw electrograms to remove the QRS complex, originating from the activation of the ventricles. To this end, the time intervals of the QRS complex in the 4s clinical window were determined based on the ECG recordings. For each of the 64 electrodes, the electrogram morphology during these intervals was then averaged to obtain an average ECG morphology. This morphology was then subtracted from the raw electrogram, resulting in an electrogram devoid of artifacts from ventricular activation. Next, we applied a 1.5–25 Hz fourth-order Butterworth band pass filter and computed the dominant cycle length, T_CL_, for each channel from the Welch Power Spectrum Density estimate. Using T_CL_, we constructed a so-called recomposed signal. This signal was composed of a sum of single-period sinusoidal waves with a period equal to T_CL_ and a magnitude equal to the absolute value of the slope of the electrogram when this slope is negative or equal to zero when this slope is positive. As further detailed in Ref. [33], the final recomposed signal is the sum of all these single-period waves. Importantly, this signal has a sinusoidal morphology oscillating around zero and its zero crossings are identifiable as the electrode’s activation time. Once this procedure was carried for all time points in the electrogram, we computed the phase of the recomposed signal using the Hilbert Transform and constructed phase maps. We limited ourselves to rotational activation patterns, and their number was determined by identifying spiral wave activity in each 4-second clinical mapping window that was present for ≥3 cycles [38] by 3 reviewers using a blinded assessment (AJR, MIH, MR). This methodology was used in previous work [39,40], where it was found to give an overall κ score of 0.56 (P = 0.001), indicating moderate agreement [40].

### Creation of 2D domains, 3D electrode shells, and projection onto 3D atrial shell

**Fig 1:**
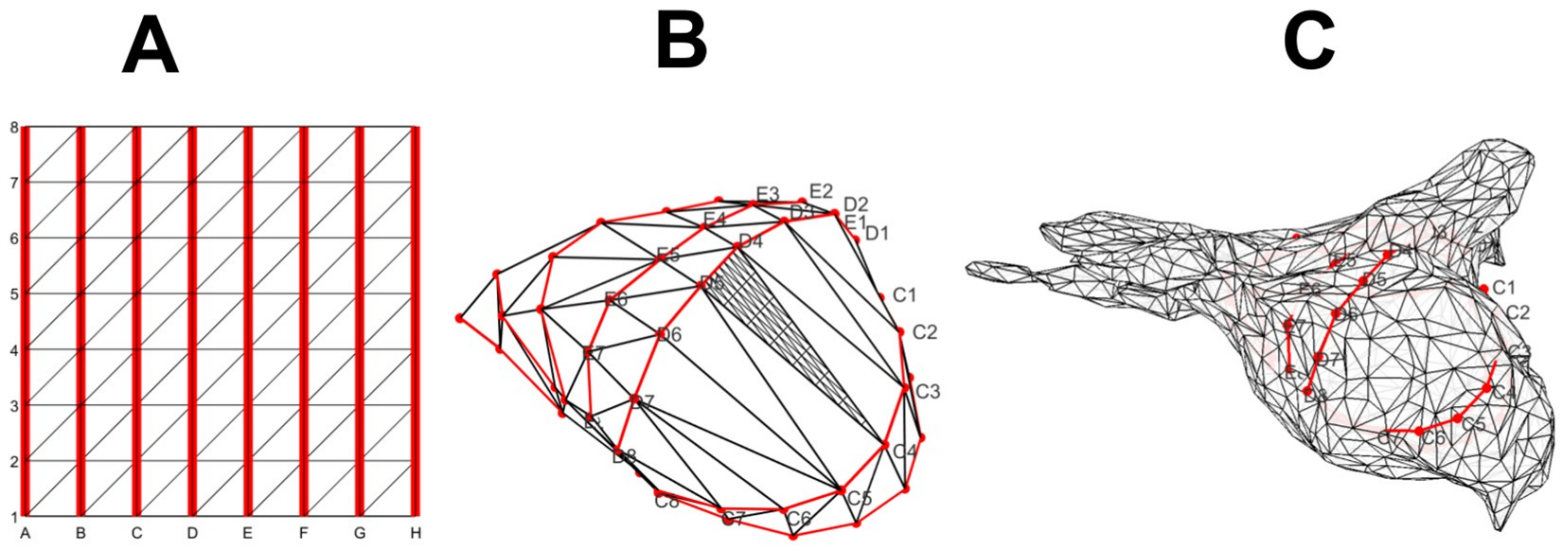
Overview of 2D and 3D geometries of AF maps. (A) Triangularized 2D grid with electrode splines highlighted in red. (B) Corresponding 3D triangularized mesh of the electrode basket, with electrode splines again highlighted in red. The triangle C4-D4-D5 shows the interpolated mesh after three subdivisions; this was omitted on other faces for clarity. (C) Corresponding atrial mesh determined from NavX or Carto, also with basket splines in red.

To visualize phase maps in 2D domains as in earlier studies [6,25,41,42], basket electrode positions are projected onto a regular, square 8x8 grid with splines indicated by A-H while the electrodes numbered 1–8 (Fig 1A). This regular grid can be trivially triangularized, resulting in a connectivity matrix that specifies the vertices for each triangular face and their connections (Fig 1A). For example, the sub-square A1B1B2A2 contains triangles with vertices (A1,B1,B2) and (A1,B2,A2).

To represent phase using 3D electrode shells, we extracted the 3D coordinates from contact basket catheters position data, resulting in 64 electrode vertices per patient. (X,Y,Z) coordinates of each electrode (as well as other sites including the ablation catheter) were exported digitally from the NavX Precision electroanatomic mapping system (Abbott, St Jude Medical, IL). The connectivity matrix for the 2D grid was applied to these 64 vertices to create a 3D triangulated surface wrapped around the basket catheter (Fig 1B) within the patient’s atrium (Fig 1C). This procedure is valid as long as the splines are not crossed. To verify this explicitly, each surface was plotted and examined manually. Finally, we subdivided each triangular face into four smaller triangles and repeated this process 3 times. As a result, the final patient-specific electrode grid meshes consisted of 3970 vertices, including the original 64 electrode vertices, and 7936 faces. An example of this procedure is presented in Fig 1B where the subdivision is shown for one arbitrarily chosen face.

For visualization purposes, we also determined the phase map on the 3D atrial shell obtained using electroanatomic mapping systems (NavX, St Jude Medical, Sylmar, CA). This shell (Fig 1C) typically contains 9866 ± 3740 (mean ± SD) vertices, on which the value of the phases computed on the electrode mesh can be projected. For this, we identified the center of the electrode mesh, using the average of all electrode locations, and created lines through this center and each vertex of the atrial mesh. This line intersects a face of the electrode mesh and the value of the phase at this face was used to construct a visualization on the atrial mesh. The principal curvatures of the 3D shells are computed using an algorithm described before [43] and implemented in Matlab (Mathworks Inc., Natick, Massachusetts) [44], and used to determine the mean and Gaussian curvature.

### Phase analysis in 2D and 3D

For 2D maps, we used bi-linear interpolation of the recomposed signal, adding 3 points between two neighboring grid points in each direction, after which the phase maps were created. For each time frame, we then quantified the location of phase singularities, corresponding to the location of the spiral wave tips, using a standard approach that computes the integral of the gradient of the phase [45–47]. We also determined the location of the tips relative to each sub-square in Fig 1A (e.g., A1B1B2A1). This resulted, after averaging over time, in a 7x7 tip density matrix $D_{i,j}^{2D}$ where i,j = 1,..,7 represent the electrode number (i) and the spline (j).

In the 3D case, we used the values of the recomposed signal for each of the 64 electrode vertices and computed the signal values at each sub-triangle of the 3D mesh using barycentric coordinates λ_1_, λ_2_, and λ_3_. Specifically, the value at each sub-point within a triangular face, s_sub_, can be written as a linear combination of the values at each vertex: s_sub_ = λ_1_s_1_+λ_2_s_2_+λ_3_s_3_ with λ_1_+λ_2_+λ_3_ = 1 and λ_1_,λ_2_,λ_3_≥0. 3D phase maps were constructed using the interpolated recomposed signals and phase singularity locations were then determined as before. Just as in the 2D case, we computed a density map by counting the number of phase singularities in an area bounded by two neighboring electrodes on a spline, together with the two corresponding neighboring electrodes on a neighboring spline. This density map was then converted into a 7x7 tip density matrix $D_{i,j}^{3D}$, where entry i,j again correspond to number and spline. Each entry i,j can then be directly compared to the i,j entry in the 2D 7x7 tip density $D_{i,j}^{2D}$. To relate the entries of this matrix to the location of the electrodes within the basket we defined polar and equatorial regions: poles were defined by i = 1 and i = 7 and correspond to the space between first and second electrode of each spline (j = 1,8) and the equator of the electrode basket space, corresponding to the space between the two central electrodes of each spline, was defined by i = 4.

### Comparison between 2D and 3D data

To compare the phase data in 2D and 3D, we computed the correlation coefficient *r* between the 2D and 3D tip density map.

$$r=\frac{\sum_{i,j}(D_{i,j}^{2D}-{\overset{-}{D}}^{2D})(D_{i,j}^{3D}-{\overset{-}{D}}^{3D})}{\sqrt{\left[{\sum_{i,j}(D_{i,j}^{2D}-{\overset{-}{D}}^{2D})}^{2}][{\sum_{i,j}(D_{i,j}^{3D}-{\overset{-}{D}}^{3D})}^{2}\right]}}$$

where ${\overset{-}{D}}^{2D}({\overset{-}{D}}^{3D})$ is the spatial average of the tip density in 2D (3D). In addition, positions of phase singularities obtained in 2D maps can be directly compared to the position in the 3D shell. For this, we first determined in which triangular face the singularity was located, after which we computed its barycentric coordinates and applied it to the corresponding face in 3D. The reverse projection, from 3D to 2D, can be found in a similar way.

### Statistical analysis

Variables are expressed either as mean±standard deviation or as median (interquartile 1-interquartile 3). The Wilcoxon signed-rank test was used to compare variables.

## Results

Patient demographics are summarized in Table 1.

**Table 1:** Patient demographics.

n = 20	Summary
Age	59 ± 3 years
Female Gender	40% (8)
Non-Paroxysmal AF, % (n)	70% (14)
Duration of AF, Months	78 ± 25
BMI, kg/m^2^	28.3 ± 1.3
Hypertension, % (n)	50% (10)
Hyperlipidemia, % (n)	35% (7)
Coronary Artery Disease, % (n)	10% (2)
Diabetes, % (n)	15% (3)
CHA_2_DS_2_-VASc	1.8 ± 0.4
LV Ejection Fraction, %	58.5 ± 9.1
LA/RA recordings (n)	16/4
LA Volume Index, mL/m^2^	35 ± 10.5

### Maps of AF activation in 2D and 3D

**Fig 2:**
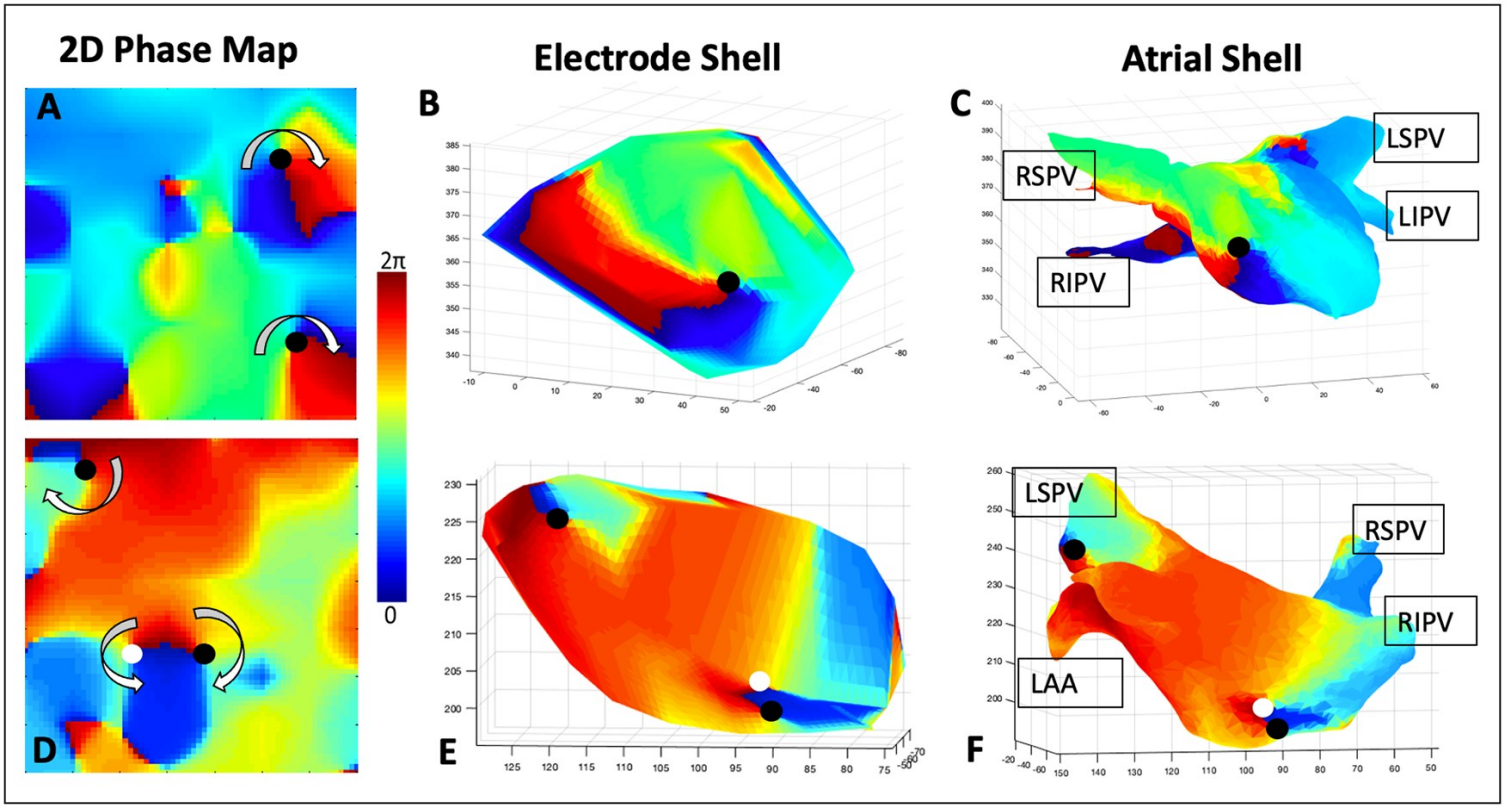
Phase maps features are conserved between 2D and 3D. (A) Snapshot of the 2D phase map in a 56 year old man with persistent AF showing a clockwise and counterclockwise rotational circuit. The phase singularities, corresponding to the tips of the spiral waves, are labeled in black (clockwise) and white (counterclockwise). (B) 3D electrode phase map corresponding to the timepoint in (A), showing the two rotational patterns seen in the 2D map. (C) Phase map projected onto the patient-specific anatomical atrial shell, along with the location of the spiral wave tips. (D-F) as in (A-C) showing three rotational circuits in a 71 year old man with persistent AF. (RSPV: Right superior pulmonary vein, RSPV: Right inferior pulmonary vein, LAA: Left atrial appendage, LPVs: Left pulmonary veins, LSPV: Left superior pulmonary vein).

The patients in this study had an average of 1.7±0.8 phase singularities during AF, similar to what we have reported before [42]. Of these, 21.6% were in the RA and 78.4% were in the LA. Fig 2A shows the 2D phase map of the left atrium of a 56 year old man during persistent AF, which reveals one clockwise (black circle) and one counterclockwise (white circle) rotational site. Electrograms for the electrodes surrounding the tip location are shown in S1 Fig. The phase map computed using the 3D electrode shell at the same instant is shown in panel B. By convention, 2D maps display endocardial surface activation while 3D maps are viewed from outside the atrium and show the epicardial surface. Fig 2 shows 3D maps of patients with persistent AF that conserve features in the 2D map. Fig 2B indicates 2 rotational sites of opposite chirality in a 70 year old man with persistent AF, which were also present on the phase map projected onto the atrial electroanatomic shell (Fig 2C).

Fig 2D–2F show another example of conservation of AF phase singularity between 2D and 3D phase maps in a 71 year old during persistent AF. The 2D map depicts two rotational sites with opposite chirality, as well as an additional isolated singular rotational site in the upper left quadrant. These features are conserved on both the 3D electrode shell (Fig 2E) and on the map projected onto the 3D electroanatomic atrial shell (Fig 2F). As expected, 3D depictions can used to locate sites with respect to relevant anatomy, and in Fig 2E the position of the potential AF rotational site is close to the left superior pulmonary vein.

### Quantitative comparison of phase mapping in two- and three-dimensions

**Fig 3:**
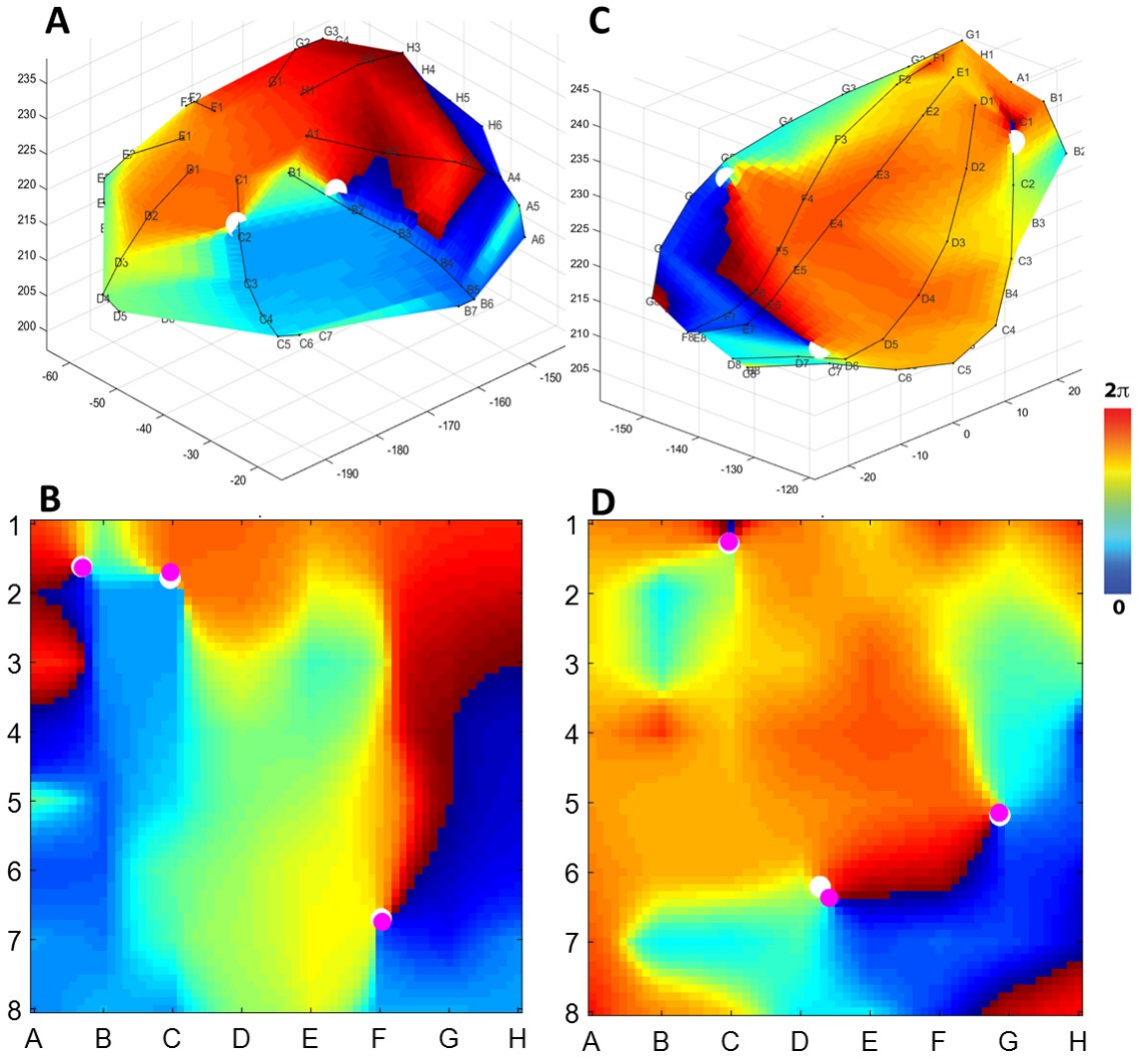
Rotational activation is conserved between 2D and reconstructed 3D shells. (A) Snapshot of a phase map of the left atrium for a 30 year old man using the electrode grid mesh showing two phase singularities (white circles). (B) Phase singularities calculated using the 2D phase map (pink circles) and using the 3D phase map, projected onto the 2D grid. (C) As in (A), and (D) as in (B), showing the right atrium for a 47 year old woman.

To provide quantitative comparisons between 3D and 2D phase maps during AF, we first computed phase singularities and their locations using 3D phase maps (see Methods). We then displayed these singularities onto the square 2D geometry and compared these projected locations to the locations of the phase singularities computed using the 2D maps. Examples of this procedure are shown in Fig 3 where we show snapshots of the electrode shell and 2D phase maps for AF in a 30-year-old man (A-B). Panel A shows that 3D phase calculations resulted in 2 visible phase singularities, labeled as white circles. In panel B, the locations of these singularities are projected back to the coordinates of the 2D map and can be seen to coincide with the locations of the phase singularities found using the 2D map (pink circles). An additional 3D phase singularity, located close to F7, was not visible in panel A but also coincides with a phase singularity found using the 2D map. Fig 3C and 3D shows this same process for AF in a 47 year old man: 3 phase singularities resulting from calculations on the 3D electrode shell are shown as white circles in panel C and are projected into 2D map coordinates in panel D. Again, these 3 phase singularities coincide with phase singularities calculated from the 2D map (pink circles). In both patients, the number of singularities in 2D and 3D is identical while their 3D locations were within less than one electrode spacing of the location of the 2D singularities, as shown by panels B and D.

**Fig 4:**
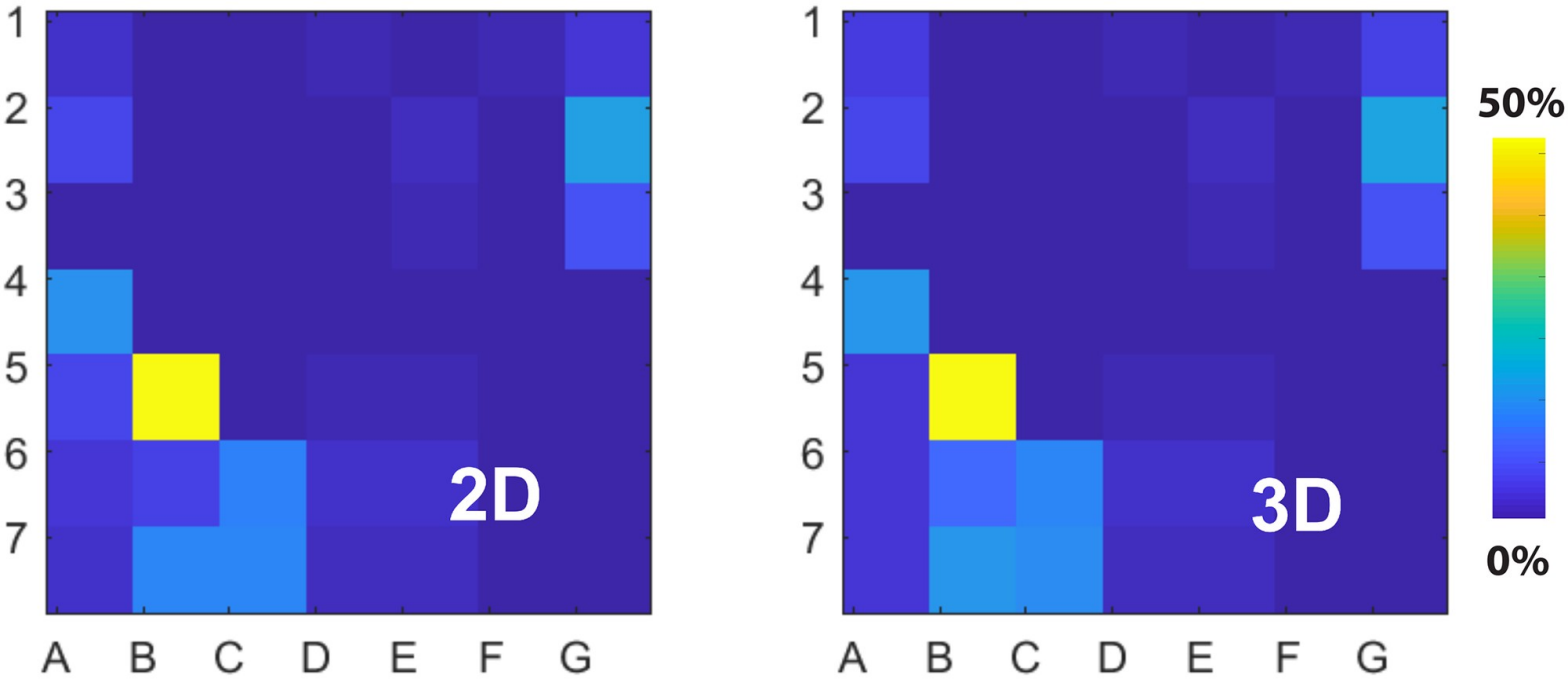
Tip density maps are conserved between 2D and 3D. The tip or phase singularity density map computed using 2D phase maps (left) and 3D maps, projected onto 2D (right) for the patient of Fig 2A–2C. Both maps are virtually identical with a correlation coefficient of r = 0.994. Percent values represent the proportion of frames during which a given electrode grid contained a tip.

**Fig 5:**
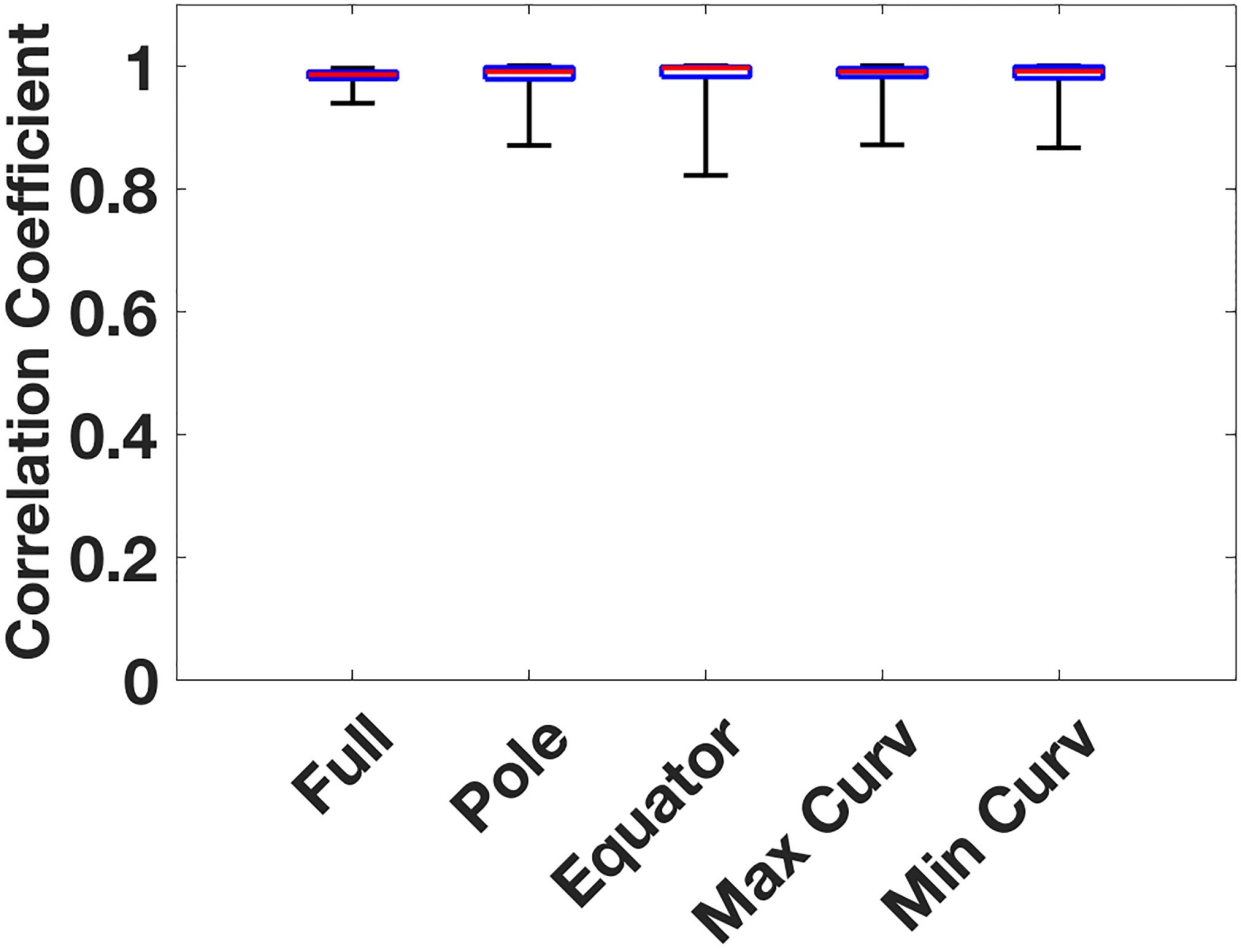
Correlation coefficient of 2D and 3D tip density does not depend on tip location or shell curvature. Plotted are, for all patients, the correlation coefficient between the 2D and 3D tip density maps for all electrodes (Full), electrodes located at the poles of the basket (Pole), the equator (Equator), and for the 7 electrodes with maximal (Max Curv) and minimal (Min Curv) mean curvature. The red bar represents the median value for each data set.

For each patient, we determined the location of 3D and 2D singularities and computed the average tip density on the regular 2D grid (see Methods). An example of the results is provided in Fig 4 where we show the 2D and 3D density maps corresponding to the patient of Fig 2A–2C. The density map in 2D and in 3D are almost indistinguishable. A quantification of this agreement can be obtained by computing the 2D correlation coefficient (see Methods). This correlation function takes on values between 0 (completely uncorrelated results) and 1 (perfect correlation). For this patient, we found that the correlation function was almost 1 (r = 0.994), indicating that the agreement between 2D and 3D was near perfect. Overall, there was excellent agreement between 2D and 3D density maps, with a median correlation coefficient of 0.985 (0.978–0.990) across all patients. In Fig 5 we summarize the correlation coefficients of the phase singularities in 2D and 3D density maps in AF for all patients. In addition, we computed the difference in distance of the maximum tip density in 2D and 3D. For this, we determined the distance between the location of the maximum tip density computed using the square 2D grid and maximum tip density computed in 3D and projected onto the 2D grid. We found an average value of 0.39±0.05 in units of electrode spacing, which results in a difference of 1.6–2.3 mm for electrodes along the spline.

### Relationship to atrial location

To determine potential effects in singularity location relative to the basket geometry, we also computed the 2D/3D correlation coefficient *r* for the polar and the equatorial region of the basket (see Methods). For the polar region, we found that the median correlation coefficient is *r* = 0.990 (0.978–0.997)) while for the equatorial region we found *r* = 0.996 (0.982–0.998; Fig 5). Importantly, these near perfect values were not significantly different from the values of *r* for the entire basket (p>0.1).

In addition, we studied the effects of the curvature (both Gaussian and mean curvature) of the atrial shell on the correlation between 2D and 3 maps. For this, we computed the 2D/3D correlation coefficient for the 7 highest curvature points in the reconstructed atrial shell as well as the 7 lowest curvature points. The median correlation coefficient for both the highest mean and Gaussian curvature points was again found to be very close to one: *r* = 0.990 (0.981–0.996). For the lowest curvature points, this value was *r* = 0.991 (0.979–0.999) and *r* = 0.991 (0.979–0.999), respectively (Fig 5). A comparison between these values revealed that neither Gaussian nor mean curvature had a significant impact on the 2D/3D correlation coefficient (p>0.1). Finally, we compared persistent and paroxysmal patients and found that the correlation coefficient for both cases was close to one (*r* = 0.982 vs. *r* = 0.988; p>0.1). Also, the correlation coefficient for RA and LA patients was almost the same and nearly unity: r = 0.987 for RA patients and r = 0.983 for LA patients (p>0.1).

## Discussion

We developed a method to visualize activation patterns of AF on curved geometries by creating tools to reconstruct a volumetric shell based on data at known 3D atrial coordinates separate from commercial 3D electroanatomic systems. We rendered the 3D shell as a collection of triangles and used barycentric coordinates to interpolate values within these triangles. Identifying the correspondence between vertex locations in 3D and in 2D representations was used to map 3D results onto 2D geometries or vice versa. This is the first demonstration of the spatial correlation between phase singularities located on a 3D map with those located on a 2D projection. Further quantification of spiral tip density in phase maps showed that sites of rotational activity were conserved between 2D and 3D, with correlations nearing one and variations attributed to slight differences in the interpolation of recomposed signals on Cartesian (2D) vs. barycentric (3D) coordinates. Neither the curvature of the 3D shell nor the basket geometry affected conservation. These results provide confidence in using 2D presentations to represent 3D activity, including complex fibrillatory patterns, as long as they do not distort the relative locations of electrodes.

### Two and three dimensional representations of AF maps

It has been suggested that 2D phase maps constructed from basket electrode recordings and projected onto 3D atrial surfaces show different activation patterns than the corresponding 3D maps [28]. This discrepancy in activation patterns [48] would be surprising since rotational activity on a 2D grid will be preserved when that grid is projected onto a curved 3D surface, unless the relative position of electrodes is changed. Consider, for example, a 2x2 square sub-grid of electrodes in 2D that activates consecutively in a rotational pattern. Projecting this sub-grid onto a 3D shell corresponds to a smooth deformation of the grid onto a curved surface. The consecutive activation sequence within this project grid will be preserved, even when the electrode spacing is not uniform. We further verified that the electrodes maintained their relative positions. Although it is difficult to pinpoint the exact reasons for prior reported discrepancies, it was suggested that improper interpolation may explain these errors [49]. Since phase is not a scalar but a circular quantity representing the angle of a complex phase vector, interpolation must be conducted using the recomposed signal or using complex phase vectors and incorrect interpolation can obscure rotational patterns [26].

### Mechanistic implications

Optical mapping of human AF [50] and mapping studies at ablation [2,7,15–21] provide evidence for spiral wave activity during human AF. Recent work has shown a significant correlation between high-resolution optical mapping of AF in human atria and clinical mapping [51]. Other studies have shown that sites of rotational electrical activity in the fibrillating heart produce mechanical vortices [52], further supporting that they represent functional and hence mechanical activity of the heart and not artifact.

Nevertheless, some studies of AF ablation of sites of rotational activity have been negative [53–55] despite overall promise in meta-analyses [22,56]. The reasons for this variability in clinical results are unclear and likely multiple, but may reflect some discrepancies between clinical mapping [39] and optically mapped human AF [57], variations and inaccuracies in map interpretation and hence ablation guidance, and varying mechanisms between patients. Projection errors onto the atrial surface have also been cited. In terms of delivering therapy, no mapping method prescribes the optimal approach to ablation. Interestingly, PVI+phase singularity ablation alone provided 77.7% single procedure success in persistent AF patients in the REAFFIRM trial, compared to 65.5% success from PVI alone (p = 0.09). This is hypothesis-generating only [24], as the trial was not powered to assess 4 treatment groups resulting from additional off-protocol ablation of complex electrograms or linear lesions that reduced success in those limbs [24]. Again, interestingly, excessive ablation of fractionated electrograms and linear lesions also reduced the success of PVI in the STAR-AF2 trial [58].

This study shows that as long as the relative positions of the electrodes are unchanged, quantitative metrics computed in 2D faithfully represent those calculated directly in 3D. This means, for example, that computations of domain sizes can be first performed using 2D maps, where they may be more easily carried out. Actual domain sizes can then be obtained by projecting the domain size found in 2D onto the patient-specific electrode shell. Likewise, angular velocity of rotational patterns and spiral waves can be first computed in 2D, after which the results can be carried over to 3D. Conversely, our results imply that a 2D representation of the propagation in 3D does not alter the qualitative features of AF dynamics.

Even though 2D maps appear to be able to guide ablation, they obscure potentially useful information. For example, determining AF organization using 3D electrode shells provides more information on the location of phase singularities relative to important anatomical structures, which can be used in future studies.

### Limitations

Although prospective ablation was performed based on a proprietary mapping algorithm, this study used freely available software (downloadable at our website) without proprietary mapping. Our mapping technique utilized a recomposed signal that was obtained assuming a fixed cycle length for the whole AF episode. For episodes in which the cycle length, e.g. during AF termination, this approach will result in sub-optimal recomposed signals. For AF episodes, however, we have recently demonstrated that it is able to identify stable rotational activation with equal accuracy as an independent mapping technique [37]. The basket contact-mapping catheter has limited resolution, but is sufficient to record organized rotational/focal activity [59,60] and has been shown by others to be superior to prior methods [61]. It remains unclear which method for detecting AF phase singularities is optimal, but a recent meta-analysis supports the use of phase mapping for AF [62].

## Conclusions

We demonstrate that activation patterns in AF were conserved between 2D and 3D at sites of termination by ablation, by creating novel tools including reconstruction of a volumetric shell and 3D phase analysis based on known 3D location data. Our tools provide a platform to quantitatively compare AF maps between the large number of emerging AF mapping techniques, and to examine the relationship of organized features in AF to natural geometric curvatures of the atrium including junctions with the pulmonary vein antra and the vena cavae.

## Supporting information

S1 Fig Electrograms surrounding the tip location. Electrograms surrounding the 4 activation maps presented in the main text (Figs 2 and 3) are shown, along with a surface lead. The red line indicates the sequential activations and demonstrates rotational activity around the phase singularity.
(DOCX)
