# Supplementary material for: Three dimensional reconstruction to visualize atrial fibrillation activation patterns on curved atrial geometry
Source: PLoS One. 2021 Apr 9;16(4):e0249873. doi: 10.1371/journal.pone.0249873 (PMC8034734; doi:10.1371/journal.pone.0249873)
Supplement: S1 Fig — Electrograms surrounding the 4 activation maps presented in the main text (Figs 2 and 3) are shown, along with a surface lead. The red line indicates the sequential activations and demonstrates rotational activity around the phase singularity. (DOCX) [file pone.0249873.s001.docx]

**Supplementary Material**

**Three Dimensional Reconstruction to Visualize**

**Atrial Fibrillation Activation Patterns on Curved Atrial Geometry**

Ricardo Abad, Orvil Collart, A.J. Rogers, Prash Ganesan, Mahmood I. Alhusseini,

Miguel Rodrigo, Sanjiv M. Narayan, Wouter-Jan Rappel


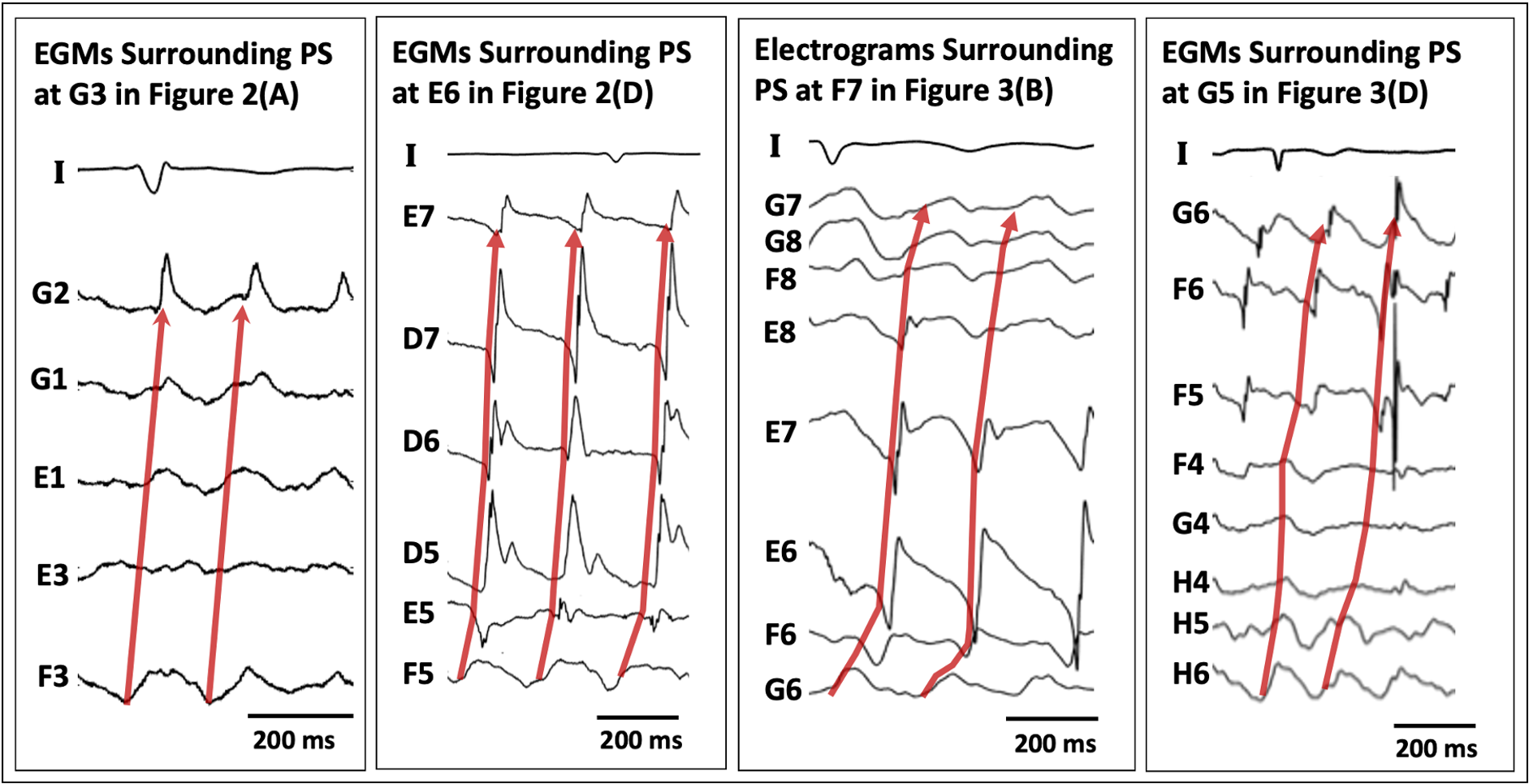


**Figure S1.** **Electrograms surrounding the tip location.** Electrograms surrounding the 4 activation maps presented in the main text (Fig. 2 and Fig. 3) are shown, along with a surface lead. The red line indicates the sequential activations and demonstrates rotational activity around the phase singularity.
